# The impact of depression and anxiety on quality of life in Chinese cancer patient-family caregiver dyads, a cross-sectional study

**DOI:** 10.1186/s12955-018-1051-3

**Published:** 2018-12-13

**Authors:** Qiuping LI, Yi LIN, Yinghua XU, Huiya ZHOU

**Affiliations:** 1 Wuxi Medical School, Jiangnan University, Wuxi, Jiangsu Province China; 2 Wuxi People’s Hospital, Wuxi, Jiangsu Province China

**Keywords:** Cancer, Anxiety, Depression, Family caregivers, Patient-caregiver dyads, Chinese

## Abstract

**Background:**

Cancer and its treatment can result in psychological distress in both adults with cancer and in their family caregivers. This psychological distress acts as a significant adverse factor in patient-caregiver dyads. The study purposes included: (i) to assess anxiety and depression in adults with cancer and their family caregivers, and examine the dyadic relationship of anxiety and depression in patient-caregiver dyads; (ii) to investigate factors that may modify these relationships; and (iii) to explore the impact of anxiety and depression on patient-caregiver dyad quality of life (QOL).

**Methods:**

This was a secondary analysis of a cross-sectional study. Participants consisted of 641 patient-caregiver dyads. Participants completed a survey assessing adults with cancer-related, family caregiver-related, and family-related variables using a demographic/clinical information sheet. In addition, anxiety/depression and QOL were assessed by using the Chinese version of the Hospital Anxiety and Depression Scale and SF-12 respectively. Data were analyzed by using descriptive statistics, Pearson correlations, subgroup analysis, and the Actor-Partner Interdependence Model.

**Results:**

Nearly one-third of participants had experienced anxiety and depression. Adults with cancer and family caregivers experienced a similar degree of anxiety and depression. Correlations (r) of anxiety and depression between patient-caregiver dyads ranged from 0.25 to 0.32. Various factors influencing the anxiety and depression relationship between patient-caregiver dyads were identified, including adults with cancer-related (e.g., age, gender, marital status, level of being informed about the disease, different types of cancer and treatment), family caregiver-related (e.g., being the spouse of a patient, duration in their role as a family caregiver, and amount of time spent on caregiving each day), and family-related (family relationship pre- and post-cancer, financial burden on the family due to cancer treatment) variables. To some extent, both actor and partner effects were identified for anxiety and depression on the QOL of patient-caregiver dyads.

**Conclusions:**

Study findings call attention to anxiety and depression, as well as related factors, in patient-caregiver dyads. The underlined essential components and focus of intervention, which will be developed to decrease psychological distress and improve QOL in patient-caregiver dyads, included individual characteristics of patient-caregiver dyads, family relationship, and anxiety and depression in their counterparts.

**Electronic supplementary material:**

The online version of this article (10.1186/s12955-018-1051-3) contains supplementary material, which is available to authorized users.

## Background

Cancer is a leading cause of death worldwide, accounting for one-sixth of all deaths. Of these, approximately 70% of all cancer deaths have occurred in low- and middle-income developing countries, including China [1]. In China, it was estimated that 4.3 million new cancer cases and 2.8 million deaths occurred in 2015 [2]. It is worth noting that in China, most new cancer cases were diagnosed at an advanced stage [3]. Advanced cancer refers to cancer that has developed to an advanced stage, when curative treatment is no longer useful, the disease is assessed to be incurable, and the patient’s condition is progressively deteriorating [4].

It is well recognized that cancer and its treatment can result in psychological distress in both adults with cancer, and in their family caregivers, who are expected to provide the cancer patient with care or support [5]. This psychological distress acts as a significant adverse factor for both adults with cancer [6–8] and their family caregivers [9, 10]. As the most common presenting symptoms of psychological distress [11], anxiety and depression have been reported in adults with cancer [11–13] and in their family caregivers [14, 15], with prevalence ranging from one-third to nearly half (45%). In China, the prevalence of anxiety and depression among cancer populations was 49.69% and 54.90% respectively, which is significantly higher than in healthy populations (anxiety: 18.37%, depression: 17.50%) [16].

It has been reported that family caregivers may experience the same level as, or an even greater level of psychological distress than adults with cancer [17, 18]. Evidence indicates that rather than affecting isolated individuals - adults with cancer or caregivers - cancer instead impacts patient-caregiver dyads as a whole [17]. This brings about the shift of the primary focus of cancer care research from the individual level - adults with cancer or family caregivers - to the dyadic level of patient-caregiver dyads [19]. Thus, there is a need to discover the dyadic relationship of anxiety and depression between adults with cancer and their family caregivers.

With research beginning to shift the focus from the individual level to the patient-caregiver dyadic level [19], studies have emerged to explore anxiety, depression and related factors in the cancer context from the dyadic perspective of both adults with cancer and family caregivers [20–25]. These studies, however, were mainly focused on family caregivers’ anxiety/depression and related factors [20–22], or the specific cancer diagnosis, e.g., lung cancer [23, 24] and prostate cancer [25]. None of the studies identified explored the factors moderating the dyadic relationship of anxiety and depression between adults with cancer and their family caregivers. Further studies on the effect of diverse demographic and medical cancer populations, and diverse patient-caregiver relationships on dyads’ psychological suffering, e.g., anxiety and depression, are required.

Recognition has been growing that anxiety and depression exert an important influence on quality of life (QOL) in both adults with cancer [13, 26, 27], and their family caregivers [15, 18, 28]. Recently, the findings of a study on Chinese adults with cancer and their spousal caregivers showed that anxiety and depression acted as the strongest independent factors influencing QOL in such couples [29]. It could be concluded from the above evidence that investigating the influencing factors of anxiety and depression in patient-caregiver dyads would benefit any interventions aimed at relieving their psychological distress and improving QOL. However, the complexity of the impact of anxiety and depression on QOL, particularly from the dyadic perspective of adults with cancer and their family caregivers, remains an area worth further exploration [29].

According to the Fletcher et al. cancer-family caregiving experience (CFCE) model [19], family caregiver anxiety and depression are influenced by both adults with cancer-related and family caregiver-related factors. Adults with cancer-related factors (e.g., patient illness-related factors and care demands) and family caregiver-related factors (e.g., role and relationship, schedule and lifestyle, employment and finances) were categorized under primary stressors and secondary stressors respectively in the domain of “stress process” in the CFCE model. Considering the complexity of a family coping with cancer together, family-related variables, including the cancer patient’s relationship with family pre- and post-cancer diagnosis, and financial burden on the family due to cancer treatment, were also analyzed as potential moderating factors for the dyadic relationship of anxiety and depression between adults with cancer and their family caregivers. Thus, to achieve a better exploration of the factors moderating the dyadic relationship of anxiety/depression between patient-caregiver dyads, the above-mentioned three variables types, including adults with cancer-related, family caregiver-related, and family-related variables, were selected as potential influencing factors in the present study. In which, adults with cancer-related factors are the variables directly related to the disease, such as cancer diagnosis and cancer treatment; family caregiver-related factors refer to those variables pertaining to the family caregiving, including the family caregiver relationship with the adult with cancer, caregiving duration and intensity; family-related factors denote those variables regarding family coping with cancer together, involving family relationship pre- and post-cancer, and financial burden on the family due to cancer treatment.

To our knowledge, no studies have been conducted in Mainland China assessing anxiety/depression, QOL, and exploring factors that moderate the dyadic relationship of adults with cancer and their family caregivers. Consequently, the study purposes were to: (i) assess anxiety/depression in adults with cancer and their family caregivers, and examine the dyadic relationship of anxiety/depression between adults with cancer and their family caregivers; (ii) investigate factors that may modify these relationships; and (iii) explore the impact of anxiety and depression on patient-caregiver dyad QOL. We hypothesized that: (i) there would be a dyadic relationship of anxiety and depression in patient-caregiver dyads; (ii) factors that moderate this relationship would include adults with cancer-related, family caregiver-related, and family-related factors; and (iii) anxiety and depression would predict QOL in patient-caregiver dyads. This study not only sheds light on the mutual impact and predictors of anxiety and depression in dyads of Chinese adults with cancer and their family caregivers, but also provides evidence for developing interventions to relieve their psychological distress and improve QOL.

## Methods

This was a secondary analysis of a cross-sectional study that evaluated the psychometric properties of the Chinese version of the Hospital Anxiety and Depression Scale (C-HADS) in dyads of Chinese adults with cancer and their family caregivers [30]. The data were collected in multiple centers (one hospital in each of the seven administrative regions in Mainland China), by convenience sampling from October 2014 to May 2015. Participants consisted of 641 dyads of adults with cancer and their family caregivers. Eligible participants met the following criteria: (i) dyads of adults with cancer and family caregivers; (ii) participant age is equal to or greater than 18 years old; (iii) the adults with cancer were diagnosed with any type of cancer, and family caregivers were identified by the adults with cancer themselves after they have provided their written informed consent; and (iv) family caregivers consented to take part in the study. Ineligibility criteria were: (i) adults with cancer suffering from other diseases, e.g., dementia, which could lead to unconsciousness and impede them from participating in the survey; (ii) those incapable of communicating in Mandarin, and (iii) who did not agree to participate in the study. Those consented or agreed to participate in the study means that participants gave their informed consent. Table 1 presents the participant characteristics.

**Table 1:** Adults with cancer and family caregiver characteristics (*n* = 641)

Characteristics	AC [n (%)]^a^	FC [n (%)]^a^
Age (mean ± SD), years	54.6 ± 12.9 (ranging from 18–88)	46.6 ± 13.2 (ranging from 18 to 79)
< 30 years	22 (3.4)	84 (13.1)
30 ~ 45 years	129 (20.1)	220 (34.3)
46 ~ 60 years	254 (39.6)	210 (33.9)
> 60 years	224 (34.9)	105 (16.4)
Gender		
Male	321 (50.1)	311 (48.5)
Female	318 (49.6)	329 (51.3)
Marital status		
Married	586 (91.4)	571 (89.1)
Not married^b^	55 (8.6)	69 (10.8)
FC relationship with adults with cancer		
Spouse		343 (53.5)
Offspring		215 (33.5)
Parent		20 (3.1)
Sibling		41 (6.4)
Other		21 (3.3)
Education levels		
Primary school or less	356 (55.5)	256 (40.0)
High school	198 (30.9)	222 (34.6)
University or above	85 (13.3)	159 (24.8)
Working status		
Working	373 (58.2)	411 (64.1)
Not working	263 (41.0)	227 (35.4)
Average time since diagnosis/duration in their role as a FC	13.3 ± 24.0 months (ranging from 1 to 228 months)	< 6 months: 348 (54.3)
		6 months ~ 2 years: 192 (30.0)
		> 2 years ~ 5 years: 55 (8.6)
		> 5 years: 39 (6.1)
Informed about the disease (n, percent)^c^		
Ill-informed	259 (40.4)	154 (24.0)
Partly informed	260 (40.6)	283 (44.1)
Well- informed	118 (18.4)	203 (31.7)
Time spent by FC in caring for patients/day [hours, n (%)]		< 2 h: 55 (8.6)
		2~4 h: 87 (13.6)
		> 4~6 h: 77 (12.0)
		> 6~8 h: 87 (13.6)
		> 8 h: 333 (52.0)
Type of cancer^d^		
Breast cancer	70 (10.9)	
Ovarian and cervical cancer	93 (14.5)	
Esophageal and gastric cancer	152 (23.7)	
Colorectal cancer	86 (13.4)	
Liver cancer	59 (9.2)	
Lung cancer	88 (13.7)	
Others	76 (11.9)	
Type of treatment		
Chemotherapy	277 (43.2)	
Chemotherapy +radiotherapy	114 (17.8)	
Chemotherapy+ surgery	162 (25.3)	
Chemotherapy+ others	81 (12.6)	
Family Characteristics^e^		
Relationship with their family members pre-cancer (n, percent)		
Very good	471 (73.5)	
Normal	162 (25.3)	
Poor	8 (1.2)	
Relationship with their family members changed post-cancer (n, percent)		
Improving	219 (34.2)	
No change	389 (60.0)	
worsening	31 (4.8)	
Financial burden on the family due to cancer treatment		
Serious	414 (64.6)	
Moderate	191 (29.8)	
Mild or None	33 (5.1)	

Note: *AC* Adult with cancer, *FC* Family caregivers, *SD* standard deviation;

^a^The total n does not equal 641 because of missing values;

^b^Not married includes: Divorced, Widowed, and Never married;

^c^Informed about disease by healthcare professionals. Well-informed: adults with cancer fully understood his/her condition; or the family caregiver was well informed about the disease of adults with cancer; Partly informed: adult with cancer was informed about the cancer diagnosis, but not about the severity of his/her condition; or family caregiver was partly informed about the disease; Ill-informed: adults with cancer or family caregivers had little information about the disease, and may or may not have been fully informed about the cancer diagnosis

^d^All of the adults with cancer had advanced cancer, were in stage III (*n* = 364, 56.8%) or stage IV (*n* = 277, 43.2%)

^e^The data for the family characteristics were reported by adults with cancer

### Procedures

Prior to study commencement, ethical approval was received from the Jiangnan University ethics committee (HSEARS20140701001), and access approval was gained from the related hospitals where the data were collected. All study participants were informed that participation in the study was voluntary, before their written informed consent was obtained. The oncologists at the related hospitals recognized the adults with cancer according to the inclusion criteria. Having obtained written informed consent from the eligible adults with cancer, the oncologists at the related hospitals inquired those who consented to take part in the study to specify their family caregivers. Family caregivers were then approached for their written informed consent. Eligible patient-caregiver dyads were contacted in the hospital wards when the adults with cancer were admitted to hospital for chemotherapy during the recovery stage. Adults with cancer and family caregivers completed all measurements independently.

### Measures

An author-developed information sheet on demographic/clinical characteristics was applied to collect data pertaining to the three types of potential influencing factors, including adults with cancer-related variables (such as age, gender, marital status, education level, employment status, understanding of the disease through information provided by healthcare professionals, cancer diagnosis and cancer treatment), family caregiver-related variables (such as age, gender, marital status, family caregiver relationship with the adult with cancer, education level, working status, understanding of the disease, caregiving duration and intensity), and family-related variables (such as family relationship pre- and post-cancer, and financial burden on the family due to cancer treatment). The data for the family characteristics were reported by the adults with cancer. In addition, C-HADS [31] and the Chinese version of the Medical Outcomes Study 12-item Short Form (C-SF-12) [32] were used to measure anxiety/depression, and QOL respectively.

#### C-HADS

The HADS is 14-item scale, categorized as two 7-item subscales measuring anxiety and depression on a 4-point Likert-type scale [31]. The simple summation of scores for items belonging to each scale results in the total anxiety and depression score. The total score range for each subscale is from 0 to 21, with higher scores indicating poorer outcomes. HADS scores for anxiety/depression (Anx/Dep) were further categorized as non- Anx/Dep case (0–7), a borderline case of Anx/Dep (8–10), and a definite case of Anx/Dep (11–21) [33]. Cronbach’s α for C-HADS was 0.93 (adults with cancer) and 0.92 (family caregivers) for the full scale, 0.87 (adults with cancer) and 0.85 (family caregivers) for both the anxiety and depression subscale in this sample.

#### C-SF-12

The SF-12 is a 12-item scale containing eight sub-scales measuring eight QOL domains. The eight domains further formulate two dimensions of the Physical Component Summary (PCS) and the Mental Component Summary (MCS) [32]. Scores of eight sub-scales and two dimensions were transformed and calculated according to the SF-12 (version 2) scoring manual. All scores range from 0 to 100: the higher the score, the better the QOL [34]. Cronbach’s α in this sample for the total SF-12 was 0.82 and 0.81 for adults with cancer and family caregivers respectively.

### Data analysis

SPSS version 21.0 was applied to analyze the data. Participant characteristics and outcome measures of the C-HADS were described by descriptive statistics. Pearson correlations and paired t-test were applied to analyze correlations and differences among paired variables of adults with cancer and their family caregivers. Subgroup analysis was conducted to investigate the factors that may modify the anxiety and depression relationship between adults with cancer and their family caregivers. That is to say, to examine the correlations of anxiety/depression between adults with cancer and family caregivers in different groups of predictors (subgroup analyses for the correlations). *P* < 0.05 was set as the level of significance.

The impact of anxiety and depression on QOL was explored by the Actor-Partner Interdependence Model (APIM) [35] using Amos 21.0. The APIM is a model of dyadic relationships that integrates a conceptual view of interdependence with the appropriate statistical techniques for measuring and testing it, which is considered a versatile approach to modeling dyadic data [36]. In the APIM (Additional file 1: Figure S1), an actor effect is the effect of an individual’s characteristics (e.g., anxiety of family caregiver) on their own outcomes (i.e., PCS of family caregiver), while partner effect refers to the effect of an individual’s characteristics (e.g., anxiety of family caregiver) on their partner’s outcomes (i.e., PCS of adult with cancer) [36]. Additional file 1: Figure S1 shows the theoretical APIM, which was designed to test the impact of anxiety and depression on QOL at the dyadic level. As shown, anxiety directly, and indirectly through depression, predicted on the SF-12 domains as actor effects (from A-a to A-d) and/or as partner effects (from P-a to P-d) in the 10 models, respectively. The SF-12 domains in the 10 models (Models 1–10) included the aforementioned two dimensions of PCS, MCS, and eight subscales of physical functioning, role-physical, bodily pain, general health, vitality, role-emotional, social functioning and mental health.

The covariance matrices in all 10 models were estimated using the maximum likelihood method. Three indices of Chi-Square χ^2^, a confirmatory fit index (CFI), and a root mean square error of approximation (RMSEA) were applied to evaluate the goodness of fit of the models. The level of a good model fit was set at *P* > 0.05 in the χ^2^, a value of CFI ≥ 0.95, and RMSEA ≤0.08 [37]. The missing data were addressed using excluded cases analysis by analysis.

## Results

Participant characteristics are presented in Table 1. Of the 641 patient-caregiver dyads, the mean age of the adults with cancer (54.6 years) was higher than that of their family caregivers (46.6 years). Most participants were living with their spouse, and over half of the family caregivers were spouses of adults with cancer. A larger proportion of adults with cancer (40.4%) was ill-informed by healthcare professionals about their disease, compared to family caregivers (24.0%). All adults with cancer were at an advanced stage of cancer, and were receiving chemotherapy. The majority of the adults with cancer (73.5%) had a good relationship with their family before the cancer diagnosis. Nearly two-thirds of families (64.6%) had experienced serious financial burden due to the cancer treatment.

### Relationship of anxiety and depression between adults with cancer and their family caregivers

Descriptive statistics for C-HADS are displayed in Table 2. As is shown, nearly one-third of participants experienced anxiety and depression. Pearson’s correlation coefficients between the C-HADS subscales indicated that the C-HADS subscales were positively correlated, both within adults with cancer or family caregivers as individuals, and between patient-caregiver dyads (Table 3). The findings on the paired variables correlations and differences between adults with cancer and family caregivers in C-HADS showed that the three paired variables of C-HADS total, anxiety and depression among adults with cancer and family caregivers were significantly related; and adults with cancer and family caregivers experienced a similar degree of psychological distress in terms of C-HADS total, anxiety and depression (Table 4).

**Table 2:** Descriptive statistics for the subscales of C-HADS scales in adults with cancer and family caregivers (*n* = 619–613*)

Variables	n*	percent	Minimum	Maximum	Mean	SD
Variables of adults with cancer						
C-HADS total	619	96.6	0.00	42.00	17.46	9.12
C-HADS-Anxiety	626	97.7	0.00	21.00	8.87	4.55
Non Anxiety	261	40.7	0.00	7.00	4.71	2.03
Borderline case**	158	24.6	8.00	10.00	8.96	0.84
Anxiety	207	32.3	11.00	21.00	14.06	2.85
C-HADS- Depression	631	98.4	0.00	21.00	8.57	4.95
Non Depression	267	41.7	0.00	7.00	4.01	2.18
Borderline case	166	25.9	8.00	10.00	8.91	0.82
Depression	198	30.9	11.00	21.00	14.44	2.94
Variables of family caregivers						
C-HADS total	621	96.9	0.00	42.00	17.46	8.83
C-HADS-Anxiety	628	98.0	0.00	21.00	8.85	4.48
Non Anxiety	246	38.4	0.00	7.00	4.52	2.16
Borderline case	172	26.8	8.00	10.00	8.96	0.83
Anxiety	210	32.8	11.00	21.00	13.83	2.68
C-HADS- Depression	631	98.4	0.00	21.00	8.60	4.74
Non Depression	250	39.0	0.00	7.00	4.00	2.42
Borderline case	181	28.2	8.00	10.00	8.98	0.83
Depression	200	31.2	11.00	21.00	14.00	2.66

*The total n does not equal 641 because of missing values

**Borderline case refers to their score was on the border of anxiety/depression

*C-HADS* Chinese version of Hospital Anxiety and Depression Scale, *SD* Standard Deviation

**Table 3:** Bivariate correlations of adults with cancer’ and family caregivers’ C-HADS (*n* = 603–631*)

	1	2	3	4	5
1. C-HADS total_AC	–				
2. C-HADS-Anxiety_AC	0.95^**^	–			
3. C-HADS- Depression_AC	0.96^**^	0.83^**^	–		
4. C-HADS total_FC	0.32^**^	0.30^**^	0.31^**^	–	
5. C-HADS-Anxiety_FC	0.31^**^	0.31^**^	0.29^**^	0.96^**^	–
6. C-HADS- Depression_FC	0.29^**^	0.25^**^	0.30^**^	0.96^**^	0.84^**^

*The total n does not equal 641 because of missing values in C-HADS.

**P<0.01

*C-HADS* Chinese version of Hospital Anxiety and Depression Scale, *SD* Standard Deviation, *AC* Adults with Cancer, *FC* Family Caregivers

**Table 4:** Correlations and differences between paired samples of adults with cancer and family caregivers in C-HADS (*n* = 603–623*)

Variables			AC		FC		Correlations		Differences	
		n *	Mean	SD	Mean	SD	r	*P*-value**	t	*P*-value
Pair 1	C-HADS Total	603	17.49	9.14	17.36	8.77	0.32	**<0.001**	0.30	0.764
Pair 2	Anxiety	614	8.89	4.57	8.81	4.45	0.31	**< 0.001**	0.37	0.709
Pair 3	Depression	623	8.57	4.95	8.57	4.74	0.30	**<0.001**	0.00	1.000

*The total n does not equal 641 because of missing values in C-HADS.

**P<0.01

*C-HADS* Chinese version of Hospital Anxiety and Depression Scale, *SD* Standard Deviation, *AC* Adults with Cancer, *FC* Family Caregivers

### Factors influencing correlations of anxiety or depression between adults with cancer and their family caregivers

Three types of variables were investigated for potential factors influencing correlations of anxiety and depression between adults with cancer and their family caregivers (Table 5): adults with cancer-related variables (5a), family caregiver-related variables (5b), and family-related variables (5c).

**Table 5:** Factors that moderate the correlations of anxiety and depression between adults with cancer and their family caregivers

Factors	5a: AC-related variables					5b: FC-related variables				
	n^c^	Correlation of Anx		Correlation of Dep		n^c^	Correlation of Anx		Correlation of Dep	
		r	*P*-value	r	*P*-value		r	*P*-value	r	*P*-value
Age (years)										
< 30	21	0.391	0.089	0.525^a^	0.017	83	0.414 ^b^	<0.001	0.354^b^	0.001
30 ~ 45	127	0.156	0.082	0.122	0.174	217	0.320 ^b^	<0.001	0.282^b^	< 0.001
46 ~ 60	247	0.424^b^	< 0.001	0.358^b^	< 0.001	203	0.327 ^b^	< 0.001	0.309^b^	< 0.001
> 60	218	0.312^b^	< 0.001	0.335^b^	< 0.001	103	0.308^b^	0.002	0.363^b^	< 0.001
Gender										
Male	309	0.373^b^	< 0.001	0.330^b^	< 0.001	302	0.274^b^	< 0.001	0.284^b^	< 0.001
Female	310	0.269^b^	< 0.001	0.289^b^	< 0.001	320	0.352^b^	< 0.001	0.319^b^	< 0.001
Marital status										
Married	570	0.323^b^	< 0.001	0.312^b^	< 0.001	555	0.303^b^	< 0.001	0.290^b^	< 0.001
Not married	53	0.225	0.117	0.238	0.086	67	0.486^b^	< 0.001	0.513^b^	< 0.001
FCs’ relationship with patients										
Spouses						334	0.330^b^	< 0.001	0.360^b^	< 0.001
Offspring						209	0.387^b^	< 0.001	0.335^b^	< 0.001
Parents						20	0.425	0.070	0.454^a^	0.044
Sibling						39	0.028	0.862	−0.128	0.438
Others						21	0.013	0.954	−0.025	0.916
Education levels										
Primary school or less	346	0.269^b^	< 0.001	0.231^b^	< 0.001	246	0.263^b^	<0.001	0.202^b^	0.001
High school	194	0.376^b^	<0.001	0.427^b^	<0.001	217	0.339^b^	<0.001	0.348^b^	<0.001
University or above	81	0.308^b^	0.006	0.280^a^	0.011	156	0.335^b^	<0.001	0.355^b^	<0.001
Working status										
Working	360	0.297^b^	<0.001	0.307^b^	<0.001	400	0.268^b^	<0.001	0.264^b^	<0.001
Not working	259	0.322^b^	<0.001	0.280^b^	<0.001	220	0.385^b^	<0.001	0.353^b^	<0.001
Duration in their role as a family caregiver										
< 6 months						340	0.228^b^	<0.001	0.189^b^	<0.001
6 months~ 2 years						186	0.505^b^	<0.001	0.528^b^	<0.001
> 2 ~ 5 years						52	0.257	0.066	0.300^a^	0.029
> 5 years						37	0.105	0.538	0.255	0.128
Informed about the disease										
Ill-informed	256	0.276^b^	<0.001	0.233^b^	<0.001	151	0.303^b^	<0.001	0.162^a^	0.046
Partly informed	248	0.361^b^	<0.001	0.360^b^	<0.001	273	0.319^b^	<0.001	0.332^b^	<0.001
Well-informed	115	0.149	0.112	0.173	0.065	198	0.310^b^	<0.001	0.351^b^	<0.001
Time spent by family caregivers in caring for patients / day										
< 2 h						54	0.361^b^	0.007	0.234	0.085
2~4 h						85	0.344^a^	0.001	0.391^b^	<0.001
> 4~6 h						72	0.400^b^	<0.001	0.361^b^	0.002
> 6~8 h						84	0.405^b^	<0.001	0.425^b^	<0.001
> 8 h						322	0.242^b^	<0.001	0.217^b^	<0.001
Type of cancer										
Breast cancer	67	0.376^b^	0.002	0.351^b^	0.003					
Ovarian and cervical cancer	88	0.176	0.101	0.157	0.135					
Esophageal and gastric cancer	147	0.551^b^	<0.001	0.485^b^	<0.001					
Colorectal cancer	83	0.306^b^	0.005	0.228^a^	0.038					
Liver cancer	59	0.110	0.410	0.168	0.203					
Lung cancer	85	0.203	0.062	0.295^b^	0.007					
Others	74	0.342^b^	<0.001	0.306^b^	0.008					
Type of treatment										
Chemotherapy	267	0.330^b^	<0.001	0.305^b^	<0.001					
Chemotherapy +surgery	112	0.250^b^	0.009	0.202^a^	0.035					
Chemotherapy +radiotherapy	160	0.380^b^	<0.001	0.361^b^	<0.001					
Chemotherapy+ others	80	0.207	0.067	0.270^a^	0.015					
5c: Family-related variables										
Relationship with their family members pre-cancer										
Very good	459	0.321^b^	<0.001	0.326^b^	<0.001					
Normal	157	0.252^b^	0.001	0.172^a^	0.032					
Poor	8	0.219	0.602	−0.156	0.712					
Relationship with family members changed post-cancer										
Improving	213	0.345^b^	<0.001	0.390^b^	<0.001					
No change	377	0.293^b^	<0.001	0.250^b^	<0.001					
Worsening	31	0.312	0.088	0.246	0.183					
Financial burden on the family due to cancer treatment										
Serious	400	0.316^b^	<0.001	0.346^b^	<0.001					
Moderate	187	0.232^b^	0.002	0.154^a^	0.035					
Mild or None	33	0.613^b^	<0.001	0.380^a^	0.029					

Note: *AC* Adults with Cancer, *FC* Family Caregivers, *Anx* Anxiety, *Dep* Depression, *Correlation of Anx* Correlation of Anx between adults with cancer and their family caregivers, *Correlation of Dep* Correlation of Dep between adults with cancer and their family caregivers

^a^Correlation is significant at the 0.05 level (2-tailed)

^b^Correlation is significant at the 0.01 level (2-tailed)

^c^The total n does not equal 641 because of missing values

#### Adults with cancer-related variables

Among adults with cancer-related variables (5a), age; gender; marital status; education level; employment status; level of being informed about the disease; different types of cancer and treatment were identified to have a small to moderate effect (*r* = 0.202–0.551) on the relationship between adults with cancer and family caregiver anxiety and/or depression. Non-significant correlations between adults with cancer and family caregivers, anxiety and/or depression were found in younger adults with cancer; in those who were not married; who were well-informed about the disease; had ovarian or cervical cancer; had liver cancer; had lung cancer; and were undergoing chemotherapy plus other treatments.

#### Family caregiver-related variables

Almost all family caregiver-related variables (5b), including age, gender, marital status, being the spouse or offspring of a patient; education level, employment status; shorter duration in their role as a family caregiver, level of being informed about the disease, and a longer amount of time spent on caregiving each day, were found to have a small to moderate effect (*r* = 0.189–0.528) on the relationship between adults with cancer and family caregiver anxiety and/or depression. Non-significant correlations between adults with cancer and family caregiver anxiety and/or depression were found in the adult with cancer’s parents, siblings, and others; a longer duration in their role as a family caregiver; and a shorter amount of time spent on caring for the patient per day.

#### Family-related variables

All three family-related variables (5c) included in this study were found to have a small to moderate effect (*r* = 0.172–0.613) on the relationship between adults with cancer and family caregiver anxiety and/or depression. Non-significant correlations between adults with cancer and family caregiver anxiety and/or depression were found in those who reported having a poor relationship with their family pre-cancer diagnosis; and/or the relationship with their family was worsening after the cancer diagnosis.

### Impact of anxiety and depression on QOL

The APIM analysis of all 10 sub-models (Additional file 2: Figure S2 and Table 6) showed goodness of fit with the indices of Chi-Square χ^2^ = 2.280~ 2.456, *P* = 0.284~0.320; all the CFI = 1.000; and RMSEA = 0.015~0.020. These good fits to the models of the APIM analysis support the designated theoretical view, in that anxiety and depression are interrelated and exert, to varying degrees, both actor and partner effects on the QOL of adults with cancer and family caregiver dyads.

**Table 6:** Standardized path coefficients and fit statistics of ten models

Indicates	M 1	M 2	M 3	M 4	M5	M6	M7	M8	M9	M10
Dyadic Variables	PCS	MCS	PF	RP	BP	GH	VT	RE	SF	MH
Number of distinct sample moments:	27	27	27	27	27	27	27	27	27	27
Number of distinct parameters to be estimated	25	25	25	25	25	25	25	25	25	25
Degrees of freedom	2	2	2	2	2	2	2	2	2	2
Anxiety_AC➙Depression_AC	**0.83** ^**^	**0.83** ^**^	**0.83** ^**^	**0.83** ^**^	**0.83** ^**^	**0.83** ^**^	**0.83** ^**^	**0.83** ^**^	**0.83** ^**^	**0.83** ^**^
Anxiety_FC➙Depression_FC	**0.84** ^**^	**0.84** ^**^	**0.84** ^**^	**0.84** ^**^	**0.84** ^**^	**0.84** ^**^	**0.84** ^**^	**0.84** ^**^	**0.84** ^**^	**0.84** ^**^
Anxiety _AC➙SDs_ AC (A-a)	−0.05	−0.32**	− 0.05	−0.11	− 0.06	−0.13	− 0.09	−0.19**	− 0.20**	− 0.26**
Anxiety _AC➙SDs_FC (P-a)	0.09	−0.09	− 0.04	0.02	0.14*	0.10	−0.05	− 0.02	0.02	− 0.03
Depression_AC➙SDs_ AC (A-b)	−0.28**	− 0.24**	− 0.26**	− 0.22**	− 0.28**	− 0.16*	− 0.11	−0.23**	− 0.20**	− 0.28**
Depression_AC➙SDs_FC (P-b)	**− 0.14** ^**a**^	0.05	**−**0.01	− 0.13*	**− 0.14** ^*^	− 0.08	0.06	−0.05	− 0.10	0.01
Anxiety_FC➙SDs_FC (A-c)	−0.18*	− 0.27**	− 0.18*	− 0.12	−0.37**	− 0.01	−0.05	− 0.24**	− 0.21**	− 0.29**
Anxiety_FC➙SDs_ AC (P-c)	0.06	0.04	−0.05	−0.10	0.10	0.14*	0.02	−0.01	0.08	−0.01
Depression_FC➙SDs_FC (A-d)	−0.18*	− 0.26**	− 0.13	−0.31**	0.04	−0.30**	− 0.22**	− 0.25**	− 0.16*	− 0.17*
Depression_FC➙SDs_ AC (P-d)	−0.09	− 0.07	0.02	− 0.01	−0.11	− 0.12	0.04	− 0.07	−0.10	− 0.02
Chi-square **(χ**^**2**^**)**	2.456	2.289	2.326	2.415	2.521	2.315	2.356	2.280	2.426	2.456
Probability level (*P* > 0.05)	0. 293	0. 318	0. 312	0. 299	0. 284	0. 314	0. 308	0. 320	0. 297	0. 293
a confirmatory fit index (CFI > 0.95)	1.000	1.000	1.000	1.000	1.000	1.000	1.000	1.000	1.000	1.000
RMSEA < 0.08	0.019	0.015	0.016	0.018	0.020	0.016	0.017	0.015	0.018	0.019

Note: *SDs* SF-12 Domains, *AC* Adults with Cancer, *FC* Family Caregivers, A-a, A-b, A-c, A-d, A-d stands for Actor effects; P-a, P-b, P-c, P-d stands for Partner effects; *RMSEA* a root mean square error of approximation; M1 to M10 represent ten different sub-models. M1 *PCS* Physical Component Summary, M2: *MCS* Mental Component Summary, M3: *PF* Physical Functioning, M4: *RP* Role Physical, M5: *BP* Bodily Pain, M6: *GH* General Health, M7: *VT* Vitality, M8: *RE* Role Emotional, M9: *SF* Social Functioning; and M10: *MH* Mental Health

^*^P<0.05; ^**^ *P*<0.01; ^a^ 0.05<*P*<0.06

A further analysis of the 10 sub-models found that: (1) in general, the impact of anxiety and depression on QOL was negative, with one exception relating to active effect and 16 exceptions relating to partner effects (Additional file 2: Figure S2 and Table 6). (2) There were nearly three-quarters (28/40) significant actor effects, four significant partner effects, and one borderline significant partner effect: the impact of depression in adults with cancer on family caregiver PCS (*P* = 0.058).

## Discussion

According to the three study objectives, on the basis of the study findings, the following three key issues were identified that warrant further discussion: (i) the relationship of anxiety/depression between adults with cancer and their family caregivers; (ii) factors influencing correlations of anxiety/depression in adults with cancer and their family caregivers; and (iii) the impact of anxiety and depression on QOL.

### Relationship of anxiety/depression between adults with cancer and their family caregivers

The mean scores of C-HADS-anxiety (M = 8.87) and C-HADS-depression (M = 8.57) in adults with cancer were higher than those reported in a large sample population of adults with cancer (M = 6.05 and 4.38 for anxiety and depression respectively). If using the same threshold score of 8 or above as did Smith et al., the ‘possible cases’ of anxiety [56.9% (365/641)] and depression [56.8% (364/641)] in adults with cancer in the current study were much higher than those reported by Smith et al., where the ‘possible cases’ of anxiety and depression were 33.3% and 19.8% respectively [38].

For family caregivers of adults with cancer, both the mean C-HADS-anxiety score (M = 8.85) and the rate of anxiety cases (32.8%) were similar to those reported for Gough and Hudson’s family caregivers of palliative adults with cancer (M = 8.45, rate of anxiety cases 32.1%), using the higher cutoff scores of 11 or more. However, in the current sample both the mean C-HADS depression score (8.60 vs.5.88) and the rate of depression cases (31.2% vs.12.2%) were higher than those reported by Gough and Hudson [39]. Nevertheless, the approximately one-third of the incidence of anxiety and depression in the current study was in general consistent with other studies, as described earlier, both in adults with cancer [11–13] and in family caregivers of adults with cancer [14, 15].

Study results showed that the C-HADS total, anxiety and depression in adults with cancer and family caregivers were significantly related (*r* = 0.30–0.32); adults with cancer and their family caregivers experienced a similar degree of HADS total, C-HADS-anxiety and C-HADS-depression. These findings are consistent with a meta-analysis, in that the psychological distress of adults with cancer and family caregivers was closely correlated (*r* = 0.35, *P* < 0.001), and the severity of distress experienced by both patients and their informal caregivers is of roughly equal magnitude (*P* = 0.64) [40].

The similar degree of C-HADS total, anxiety and depression for adults with cancer and their family caregivers in this study may be related to: (1) caregiving demands for family caregivers to provide both practical (“caring for”, including help with treatment monitoring, dressing, washing, cooking, and feeding) and emotional (“caring about”, including showing appreciation, listening to problems, and attempting to improve psychological well-being) support to adults with cancer [41]; (2) the fact that the adults with cancer were identified at an advanced stage, when treatment possibilities are both restricted and expensive [3]; (3) and the Chinese culture. In Chinese culture, the Confucian notion of filial piety enforces an unconditional commitment on adults to personally care for their family members [42], leading to a tendency for family caregivers to put their own needs behind those of the patient [43]. In addition, not informing patients about a cancer diagnosis also places an additional burden on family caregivers. When dealing with patients, family caregivers needed to don a mask and pretend that everything was fine in order to “protect” the patient [44].

The above findings on the anxiety and depression relationship between adults with cancer and their family caregivers could indicate mutual impacts on psychological distress in terms of anxiety and depression in adults with cancer and in their family caregivers. Other studies also reported a mutual impact on psychological distress in patient-caregiver dyads [18, 28]. These findings are a reminder that treating adults with cancer and their family caregivers as a unit at the dyadic level is crucial in improving dyads’ psychological distress or suffering, e.g., anxiety and depression.

### Factors influencing correlations of anxiety/depression in adults with cancer and their family caregivers

Multiple factors moderating the relationship between anxiety/depression in dyads of adults with cancer and their family caregivers were identified using subgroup analyses for the correlations, including the following three types of variables.

#### Adults with cancer-related variables

To begin with age, it was interesting to note that in younger adults with cancer (< 30 years and 30~ 45 years), their anxiety did not correlate with the anxiety of their family caregivers, as it did in those who were older. For depression, only in adults with cancer ages 30–45 years did depression not correlate with depression in their family caregivers, as it did for those in other age groups, either older or younger. The authors speculated that these findings may be related to the fact that younger adults with cancer are typically multi-tasking in their various roles, particularly those aged 30–45, so they might be more concerned about the impact of the cancer diagnosis and treatment on their work or other responsibilities, such as child rearing and or elder care, than participants in other age groups. However, as no similar report was identified, this still needs to be validated in future research. This finding is also a reminder that healthcare professionals should pay more attention to middle-aged participants.

Our analysis also found that the correlation of anxiety/depression between adults with cancer and family caregivers was significant in all sub-groups, in terms of the gender, education level, and employment status of adults with cancer. Further analyses on sub-group differences showed that female adults with cancer had higher levels of anxiety (*P* < 0.001) and depression (*P* = 0.003) than male adults with cancer. Significant differences in both anxiety (*P* < 0.001) and depression (*P* < 0.001) existed in adults with cancer in terms of education level, with a trend indicating that the higher the education level, the lower the anxiety and depression scores. This is in line with the findings of another study on the incidence of anxiety and depression in adults with cancer, which reported that uneducated patients were in the highest category, in terms of education level, to suffer from anxiety and depression [45]. It is assumed that patients with higher education levels can easily understand the disease and its treatment, which may benefit their coping process and improve their psychological state, e.g., anxiety and depression. A trend was identified that employed adults with cancer experiencing higher levels of anxiety (*P* = 0.079) and depression (*P* = 0.059) than unemployed adults with cancer. This may echo previous speculations that it is crucial for healthcare professionals to pay more attention to patients who are multi-tasking, juggling their various work and life roles. According to these findings, it is recommended that a future topic of study should focus on employed female adults with cancer with lower education levels.

Another factor that did not moderate the correlation between anxiety/depression in adults with cancer and that of their family caregivers, was seen in unmarried adults with cancer. It is supposed that this may be due to the fact that married adults with cancer are likely more reliant on their family members, e.g., family caregivers in this case, than are those who are not married. The same profile also found that adults with cancer who were well-informed about the disease by healthcare professionals was a factor that did not influence the correlation of anxiety/depression between patient-caregiver dyads. Further ANOVA analyses found that significant differences in both anxiety (*P* < 0.001) and depression (*P* < 0.001) existed in adults with cancer, in terms of the extent to which adults with cancer were informed about the disease, with a trend that the higher the level of being informed about the disease, the lower the level of anxiety and depression. These findings are similar to those of a report on psychological distress, e.g., anxiety and depression, in patients with colon or rectal cancer. The results showed that with proper patient management, e.g., when patients were informed and educated about their disease and treatment, their anxiety and depression levels improved during the course of treatment [46].

Furthermore, cancer type and treatment were factors moderating the anxiety and depression correlations between adults with cancer and their family caregivers. This is a reminder for healthcare professionals to treat patients as individuals, according to the type of cancer they are suffering from. A previous study also showed a need for adults with cancer to receive sufficient information about their disease to help them in making decisions about their treatment [44].

#### Family caregiver-related variables

All of the general features of family caregivers, including age, gender, marital status, education level, and employment status, were identified as factors to moderate the anxiety and depression correlations between adults with cancer and their family caregivers. These discoveries again designate a mutual impact on anxiety and depression between adults with cancer and family caregivers. The following discussion mainly focuses on the disease-related (being informed about the disease) and caregiving-related (family caregiver’s relationship with patient, caregiving duration and intensity) variables.

Regarding the extent to which family caregivers were informed about the disease, there was a trend that the higher the level that family caregivers were informed about the patient’s disease, the lower their level of anxiety and depression. However, no significant difference was found among the three groups in terms of being ill-informed, partly informed, or well-informed, as there was in the adults with cancer. Nevertheless, as a factor moderating the relationship of anxiety and depression between dyads of adults with cancer and family caregivers, properly informing cancer dyads about the disease could be an area that deserves more attention from healthcare professionals in a future study. For instance, it was reported that receiving too much information about the disease was correlated to more severe hopelessness in adults with cancer [47], which may relate to higher levels of anxiety and depression in adults with cancer.

This study disclosed that being the spouse or offspring of an adult with cancer was a factor that influenced the correlation of anxiety and depression between dyads of adults with cancer and family caregivers. It is reasonable that the closer the relationship between adults with cancer and family caregivers, the stronger the correlation of anxiety and depression between them, which may also indicate higher levels of anxiety and depression. This is indeed the case in the present sample, e.g., the further ANOVA analyses uncovered that significant differences in both anxiety (*P* < 0.001) and depression (*P* < 0.001) existed among different groups in a family caregiver’s relationship with an adult with cancer, with a trend that the levels of both anxiety and depression declined, starting from spouses, and moving on to offspring, parents, siblings, to others. This was consistent with the findings of a study that found that being the spouse of a patient was a significant factor related to more serious anxiety or depressive symptoms in bereaved family caregivers of adults with cancer [48]. Other studies also reported that being a spouse of an adult with cancer was a marker of anxiety or depression in family caregivers of adults with cancer [19, 49, 50]. This may be due to the fact that spousal caregivers are disposed to make sacrifices in taking care of their spouse, making them especially susceptible [51, 52].

In addition, the study found that family caregivers who had been in their role as a family caregiver for just a short duration had significant correlations in both anxiety and depression, with the anxiety and depression of adults with cancer. The same profile was identified in family caregivers who spent a longer time on caregiving on a daily basis. It was assumed that the shorter the duration that family caregivers had spent in their role, the more effort they needed to adjust to their role, in the process of coping with cancer together with the adult with cancer. It is reasonable to assume that the longer that family caregivers spend on caregiving, the more disruptions they experience in their regular schedule, which in turn causes more stress [53]. Indeed, evidence shows that cancer and its treatment pose challenges to such dyads, leading them to readjust and adapt their roles in terms of both family and occupation [53, 54].

#### Family-related variables

Study results showed that a good relationship with family before the cancer diagnosis, and an enhanced relationship after cancer diagnosis, were factors that moderate the relationship between adults with cancer and family caregiver anxiety and depression. These results were partly in line with a study that found that adults with cancer, who reported lower familial cohesion and higher familial conflict, were associated with higher depression scores for both adults with cancer and their family caregivers [49]. Another study also reported that both anxiety and depression in adults with cancer were predicted by the family’s avoidance of communication about the patient’s cancer and a lack of emotional support from the family [55]. These findings are a reminder that family environment and family support may impact dyads’ (both patients’ and caregivers’) anxiety and depression. This suggests that improving the quality of the family dynamic, e.g., family cohesion and relationships between family members, could be the focus of future studies on relieving dyads’ anxiety and depression.

The study findings also showed that financial burden was a factor modifying the correlations of anxiety and depression between patient-caregiver dyads. It is well known that the financial impact of cancer and its treatment can be substantial [56], even for patients with comprehensive health insurance policies [57]. This result is in line with previous studies, both in quantitative surveys [29, 58] and in qualitative interviews of dyads of adults with cancer and their family caregivers [44, 59].

Previous findings from qualitative interviews indicated that financial support from family members, particularly grown children, is a common way for couples in China coping with cancer to deal with the financial difficulties they face. The participants also proposed that government financial support is needed in order for families to handle cancer and its treatment [44]. It is expected that government financial support could help those families experiencing serious financial burden due to cancer, helping relieve their financial burden and psychological distress, e.g., anxiety and depression. In addition, reasonable programs that provide reimbursement for both the medical and nonmedical expenditures required after cancer diagnosis should be developed [57], for example, family caregiver training, especially for the elderly and for those with a lower income.

These findings on the factors moderating the relationship of anxiety/depression between adults with cancer and their family caregivers further confirms that mutual impacts on psychological distress - in terms of anxiety and depression - exist between adults with cancer and their family caregivers. The above findings are also consistent with our hypothesis, in that there is a relationship between anxiety and depression in patient-caregiver dyads; and factors that moderate this relationship include adults with cancer-related, family caregiver-related, and family-related factors.

### The impact of anxiety and depression on QOL

The good fits to the 10 sub-models of the APIM analysis not only provide evidence for the view that anxiety and depression are interrelated and exert, to varying degrees, both actor and partner effects on patient-caregiver dyad QOL, but also further support the argument that there is a mutual impact between dyads of adults with cancer and family caregivers who are in the process of coping with cancer together. The general negative impact of anxiety and depression on dyad QOL is in line with our previous findings, in that there were significant negative correlations among the C-HADS and the C-SF-12 subscales, both in adults with cancer and in family caregivers [30]. These findings are also consistent with those of other studies, which have found that anxiety and depression exert an important influence on QOL in both adults with cancer [13, 26, 27], and in their family caregivers [15, 18, 28].

Based on the present analysis, it is strongly suggested that a future intervention study from the dyadic level of patient-caregiver dyads be conducted, in order to investigate the impact of improving psychological distress, such as anxiety and depression, on QOL in adults with cancer and family caregivers. Recommendations for a future intervention study include: (1) target population: dyads of adults with cancer and their family caregivers, particularly those with lower levels of education, lower levels of being informed about the disease, lower levels of family support, serious financial burden due to cancer treatment, and severe psychological distress; (2) essential components: knowledge of the disease and treatment, skills for patients care, time management by family caregivers, and skills to improve family support. In addition, financial support is required, as discussed earlier.

### Limitations

One limitation was the cross-sectional study design; future prospective studies are required to confirm the findings of this study. In addition, the fact that study participants only included Chinese adults with cancer and their family caregivers could result in a lack of generalizability of the findings to other targeted populations in diverse cultures. Also, due to the nature of secondary data analysis, certain important variables, such as family caregivers’ self-efficacy, perceived family caregiving burden/stress, are missing in the current study when considering family caregivers’ anxiety and depression. In addition, information on a number of family-related variables (such as “relationship with family” and “financial burden due to cancer treatment”) was only solicited from adults with cancer, but not from family caregivers. The perceptions of adults with cancer and family caregivers may not be the same. Further study on the effect of diverse demographics, and systematically soliciting potentially related variables from both adults with cancer and family caregivers, is required. Moreover, although the data analysis methods applied in this study met the study aims, the multiple t-tests, correlations, and subgroup analysis may commit a type I error. Future studies employing more appropriate analyses, e.g., analyzing multiple factors together, would provide an opportunity to reduce the type 1 error rate; or using a generalized linear model to analyze the concordance in patient-caregiver dyads (odds that a caregiver would report elevated symptoms if the adult with cancer also reported above the cut-off), are highly recommended.

## Conclusion

Study findings call attention to anxiety and depression, as well as related factors, at the dyadic level in dyads of Chinese adults with cancer and their family caregivers. Further, study findings highlight the following as future study areas, in which patient-caregiver dyads could benefit from intervention programs that could be established, with the goal of relieving psychological distress and improving QOL: individual features of both adults with cancer and their family caregivers, familial affiliation and support, and anxiety and depression.

## Additional files

Additional file 1: **Figure S1.** Theoretical model in testing the impact of anxiety and depression on health-related quality of life. *Legends*: SF-12 Domains were replaced by two dimensions (Sub-Model 1–2) and eight domains of SF-12 (Sub-Model 3–10). The 10 sub-models were: Sub-Model 1: Physical Component Summary (PCS); Sub-Model 2: Mental Component Summary (MCS); Sub-Model 3: Physical Functioning (PF); Sub-Model 4: Role Physical (RP); Sub-Model 5: Bodily Pain (BP); Sub-Model 6: General Health (GH); Sub-Model 7: Vitality (VT); Sub-Model 8: Role Emotional (RE); Sub-Model 9: Social Functioning (SF); and Sub-Model 10: Mental Health (MH). A-a, A-b, A-c, A-d, A-d stands for Actor effects; P-a, P-b, P-c, P-d stands for Partner effects; P=Patients, FC=Familiar Caregivers. (DOC 110 kb)

Additional file 2: **Figure S2.** Ten sub-models (sub-model 1–10) for testing the impact of anxiety and depression on QOL. (DOC 856 kb)

## Abbreviations

APIM: Actor-Partner Interdependence Model
CFI: a Confirmatory Fit Index
C-HADS: Chinese version Hospital Anxiety and Depression Scale
MCS: Mental Component Summary
PCS: Physical Component Summary
QOL: Quality of Life
RMSEA: a Root Mean Square Error of Approximation
SF-12: the Medical Outcomes Study 12-item Short Form
