# Supplementary material for: The impact of depression and anxiety on quality of life in Chinese cancer patient-family caregiver dyads, a cross-sectional study
Source: Health Qual Life Outcomes. 2018 Dec 13;16:230. doi: 10.1186/s12955-018-1051-3 (PMC6293618; doi:10.1186/s12955-018-1051-3)
Supplement: Supplementary file 1 — Figure S1. Theoretical model in testing the impact of anxiety and depression on health-related quality of life. Legends: SF-12 Domains were replaced by two dimensions (Sub-Model 1–2) and eight domains of SF-12 (Sub-Model 3–10). The 10 sub-models were: Sub-Model 1: Physical Component Summary (PCS); Sub-Model 2: Mental Component Summary (MCS); Sub-Model 3: Physical Functioning (PF); Sub-Model 4: Role Physical (RP); Sub-Model 5: Bodily Pain (BP); Sub-Model 6: General Health (GH); Sub-Model 7: Vitality (VT); Sub-Model 8: Role Emotional (RE); Sub-Model 9: Social Functioning (SF); and Sub-Model 10: Mental Health (MH). A-a, A-b, A-c, A-d, A-d stands for Actor effects; P-a, P-b, P-c, P-d stands for Partner effects; P=Patients, FC=Familiar Caregivers. (DOC 110 kb) [file 12955_2018_1051_MOESM1_ESM.doc]

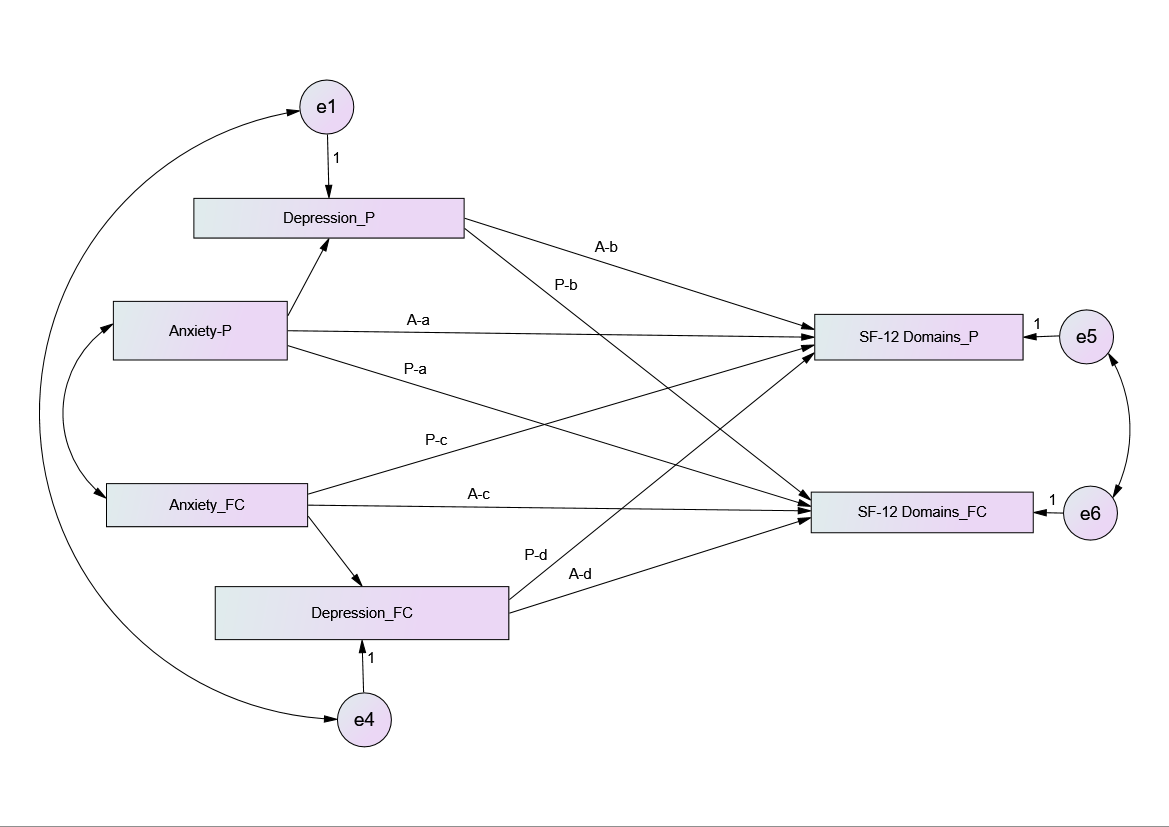


**Figure S1.** Theoretical model in testing the impact of anxiety and depression on Health related quality of life.

**Legends:**

SF-12 Domains were replaced by two dimensions (Sub-Model 1 -2) and eight domains of SF-12 (Sub-Model 3 -10).

The 10 sub-models were: Sub-Model 1: Physical Component Summary (PCS); Sub-Model 2: Mental Component Summary (MCS); Sub-Model 3: Physical Functioning (PF); Sub-Model 4: Role Physical (RP); Sub-Model 5: Bodily Pain (BP); Sub-Model 6: General Health (GH); Sub-Model 7: Vitality (VT); Sub-Model 8: Role Emotional (RE); Sub-Model 9: Social Functioning (SF); and Sub-Model 10: Mental Health (MH).

A-a, A-b, A-c, A-d, A-d stands for Actor effects;

P-a, P-b, P-c, P-d stands for Partner effects;

P=Patients, FC=Familiar Caregivers
