# Supplementary material for: The impact of depression and anxiety on quality of life in Chinese cancer patient-family caregiver dyads, a cross-sectional study
Source: Health Qual Life Outcomes. 2018 Dec 13;16:230. doi: 10.1186/s12955-018-1051-3 (PMC6293618; doi:10.1186/s12955-018-1051-3)
Supplement: Supplementary file 2 — Figure S2. Ten sub-models (sub-model 1–10) for testing the impact of anxiety and depression on QOL. (DOC 856 kb) [file 12955_2018_1051_MOESM2_ESM.doc]

**Figure S2. Ten sub-models (sub-model 1-10) for testing the impact of anxiety and depression on QOL**


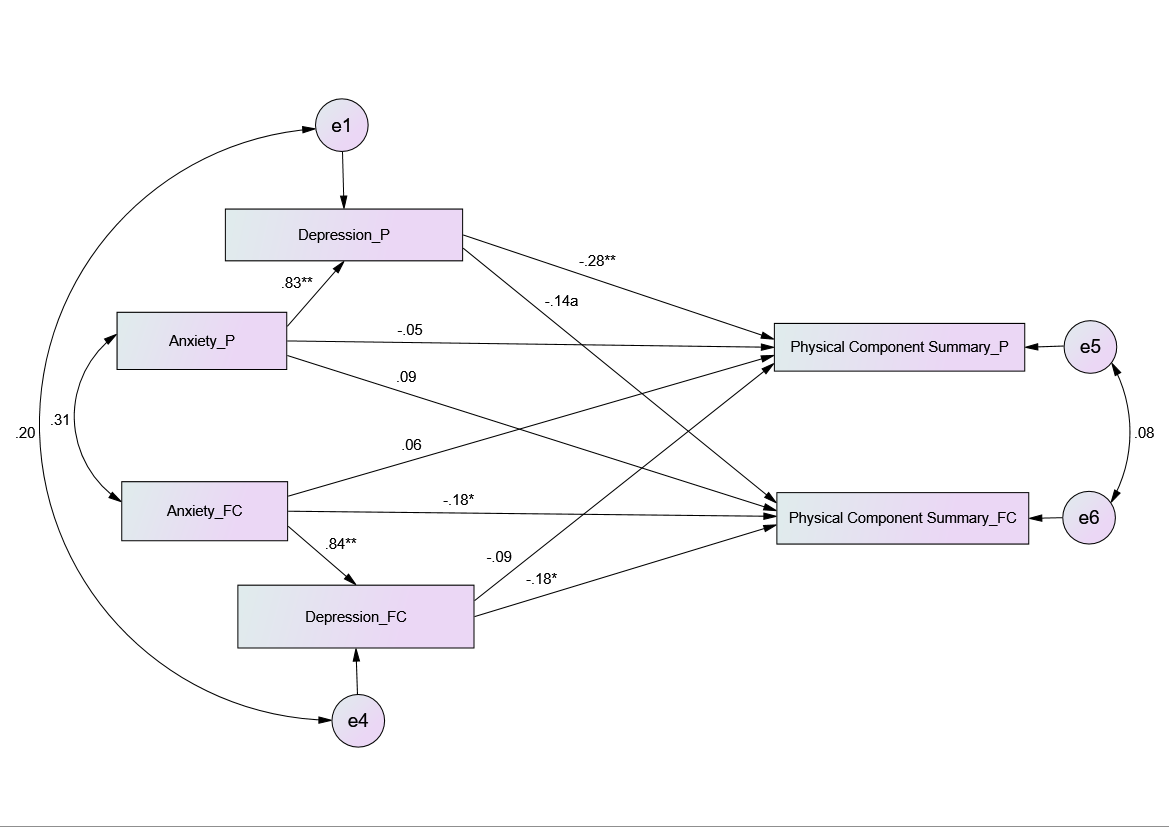


*Sub-Model 1 Physical Component Summary*

** P＜0.05; ** P＜0.01; a: P=0.058*


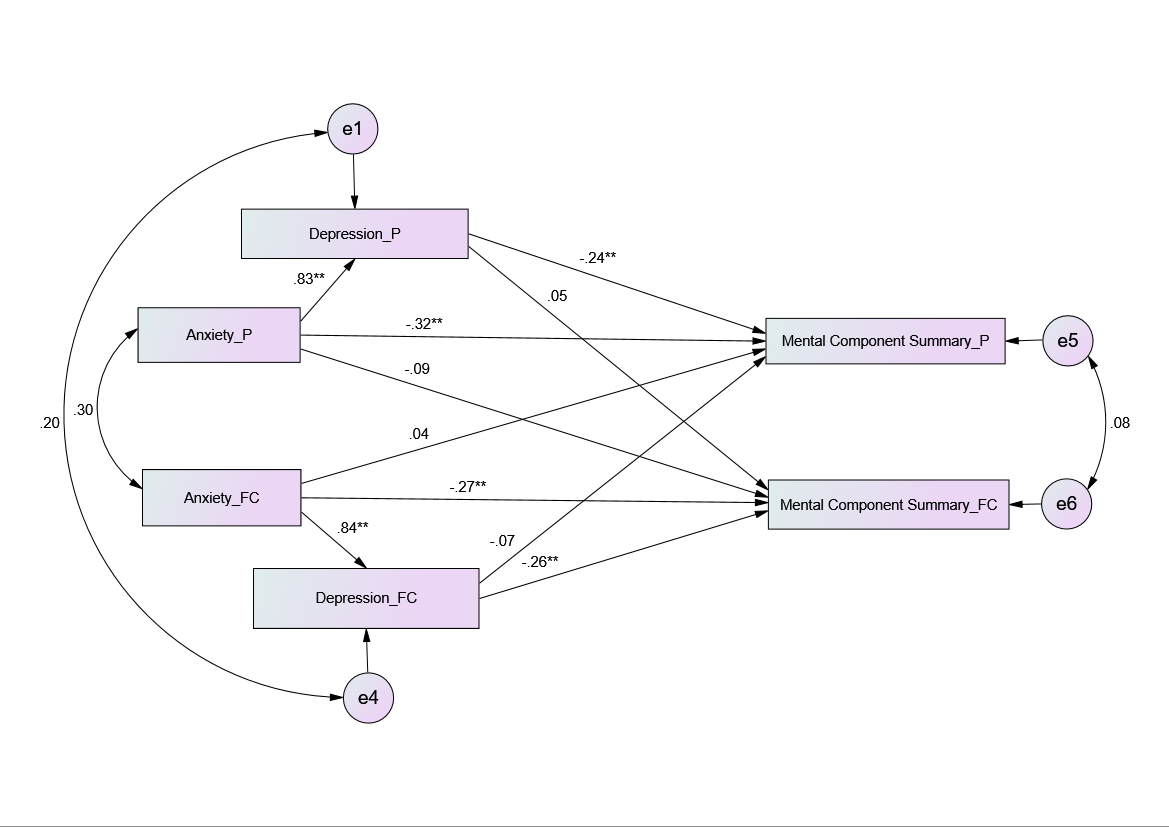


*Sub-Model 2 Mental Component Summary*

***P<0.01*

***P<0.01*


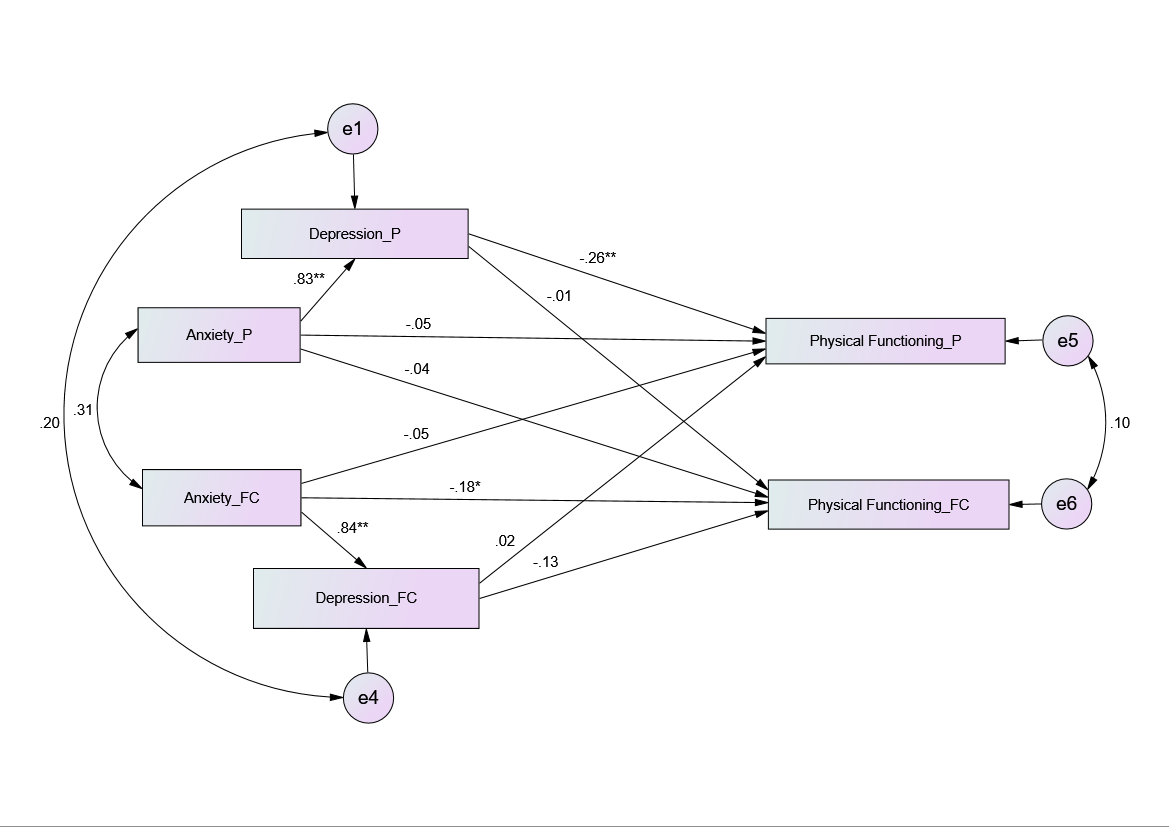


*Sub-Model 3 Physical Functioning*

**P<0.05; **P<0.01*


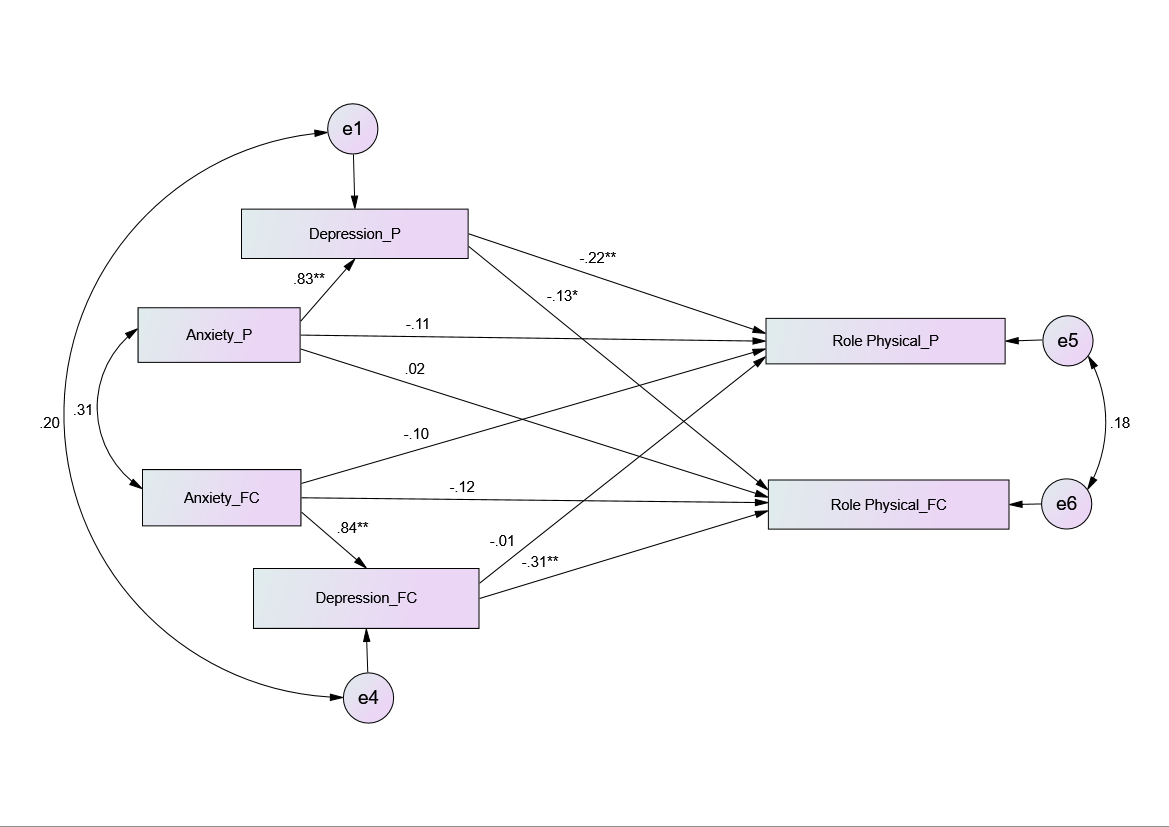


*Sub-Model 4 Role Physical*

** P＜0.05; ** P＜0.01*


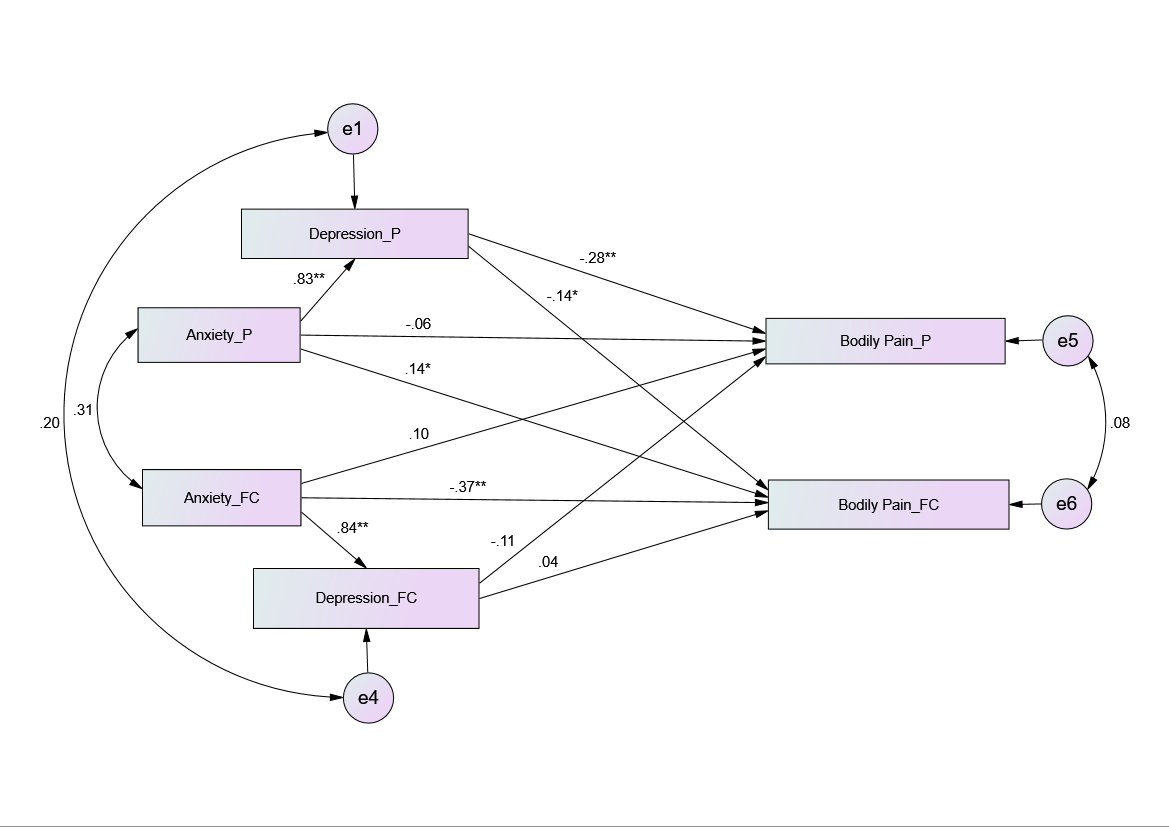


*Sub-Model 5 Bodily Pain*

** P＜0.05; ** P＜0.01*


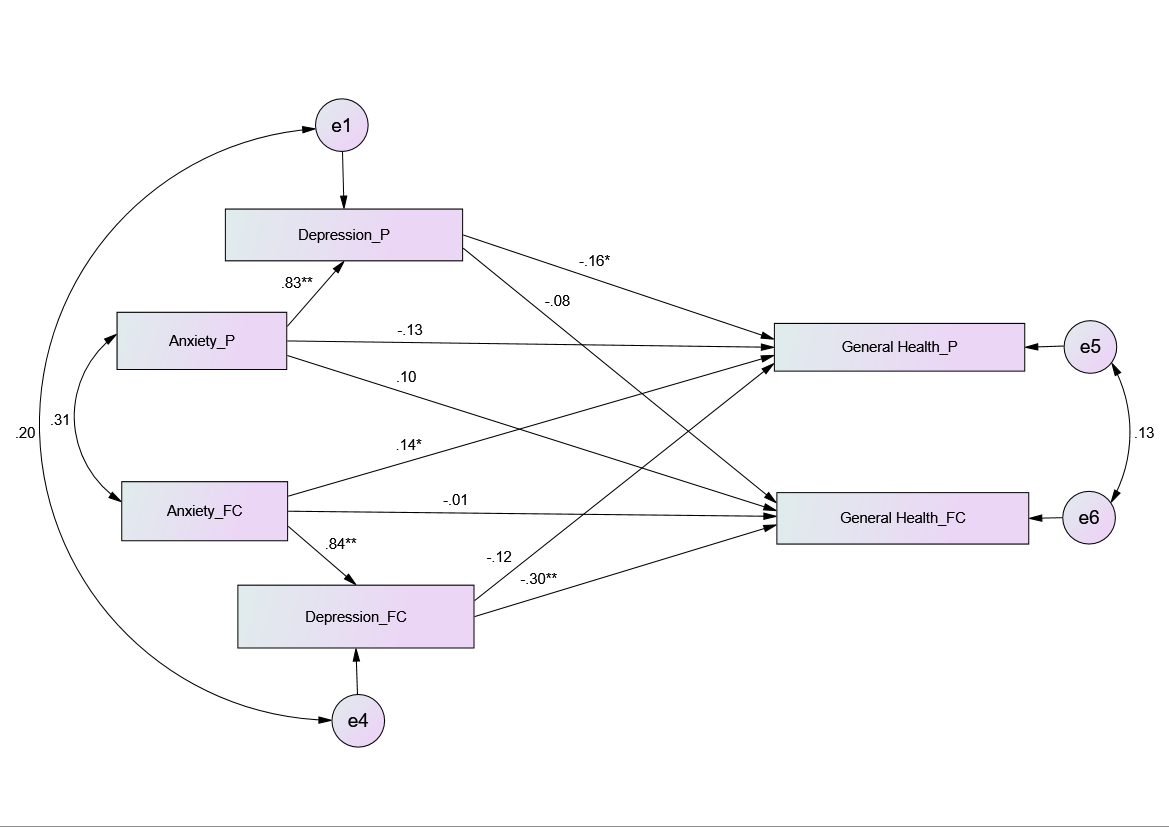


** P＜0.05; ** P＜0.01*

*Sub-Model 6 General Health*


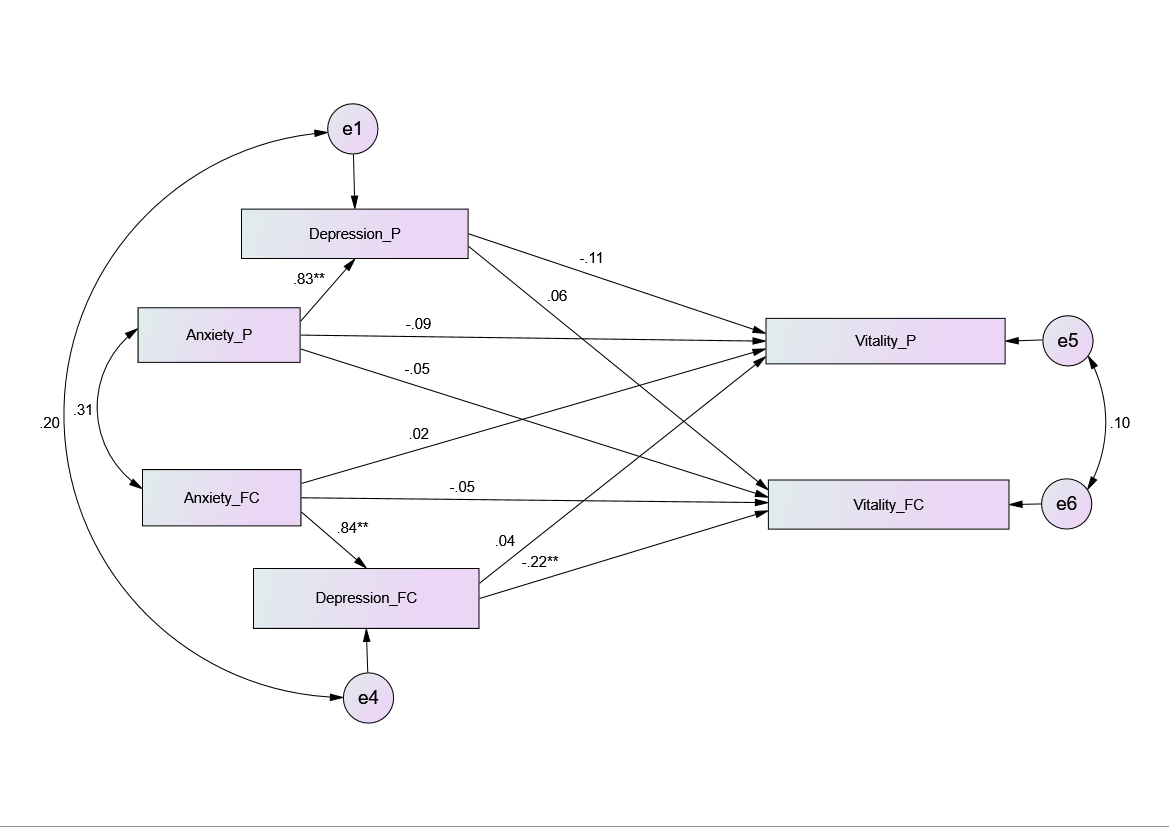


*Sub-Model 7 Vitality*

***P<0.01*


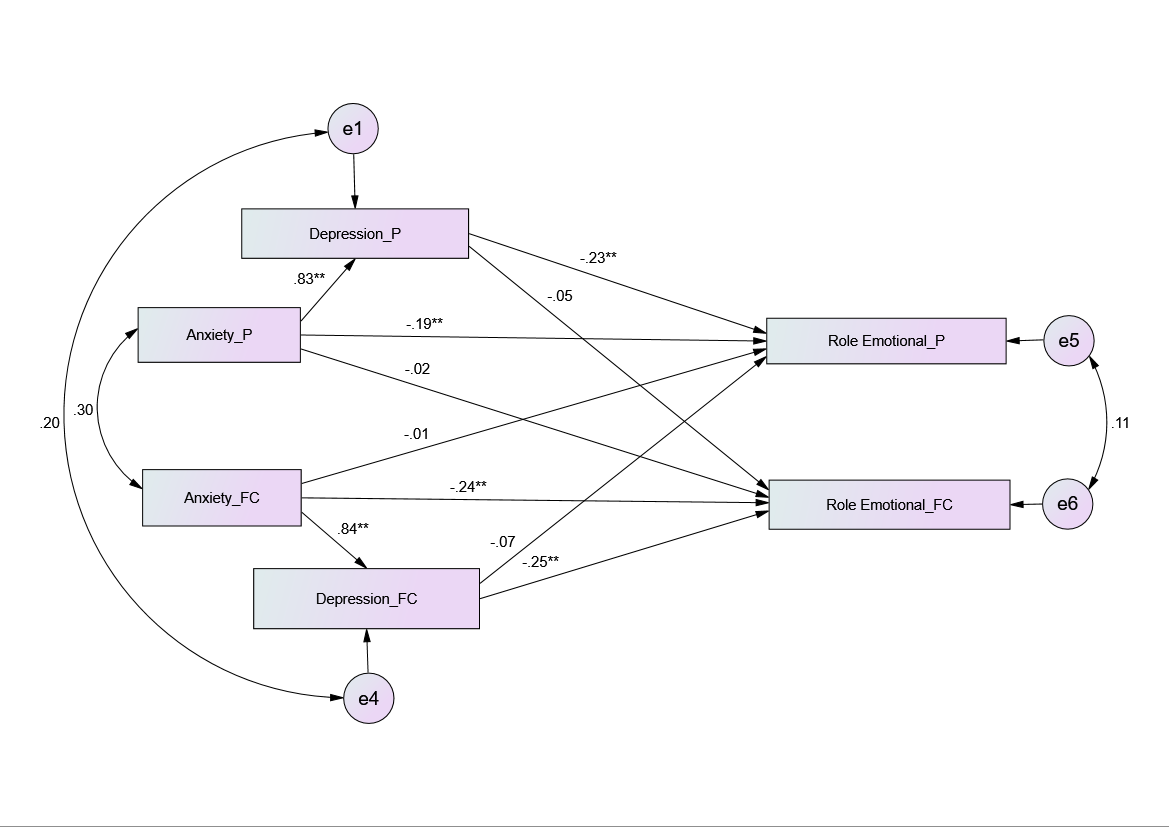


***P<0.01*

*Sub-Model 8 Role emotional*


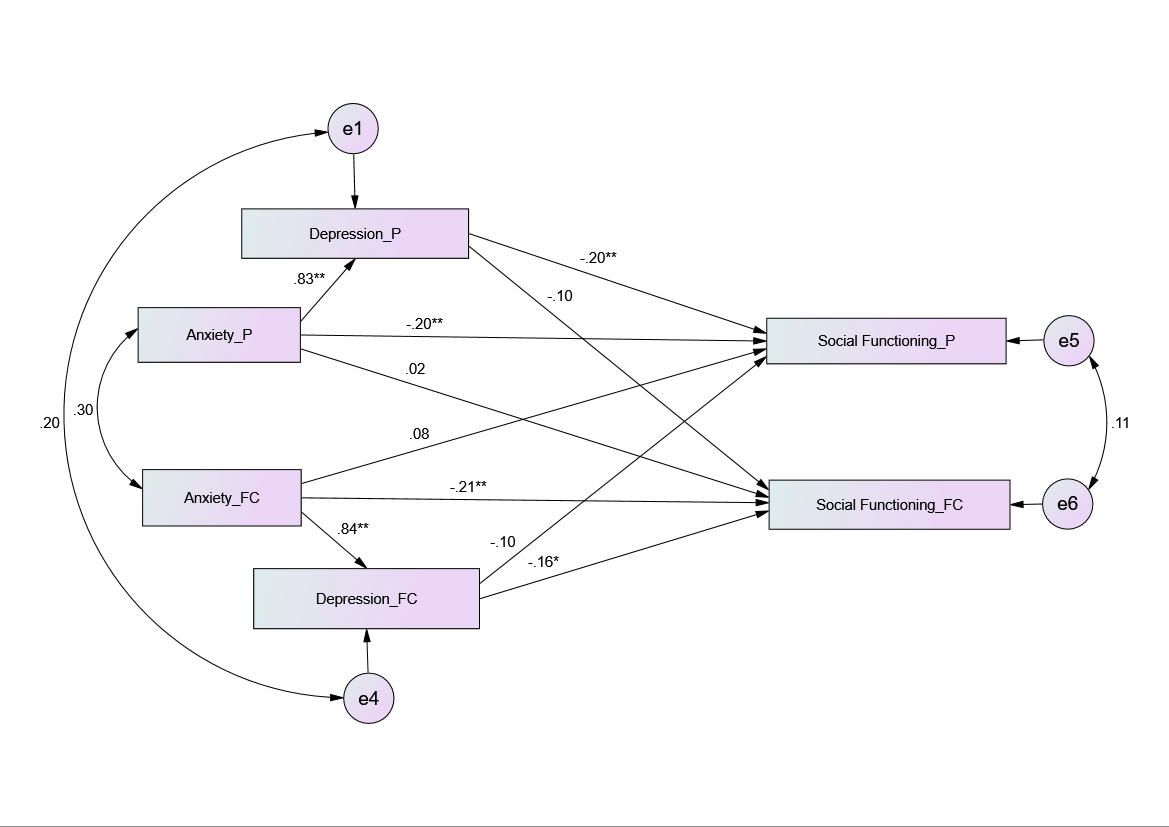


*Sub-Model 9 Social Functioning*

**P<0.05; **P<0.01*


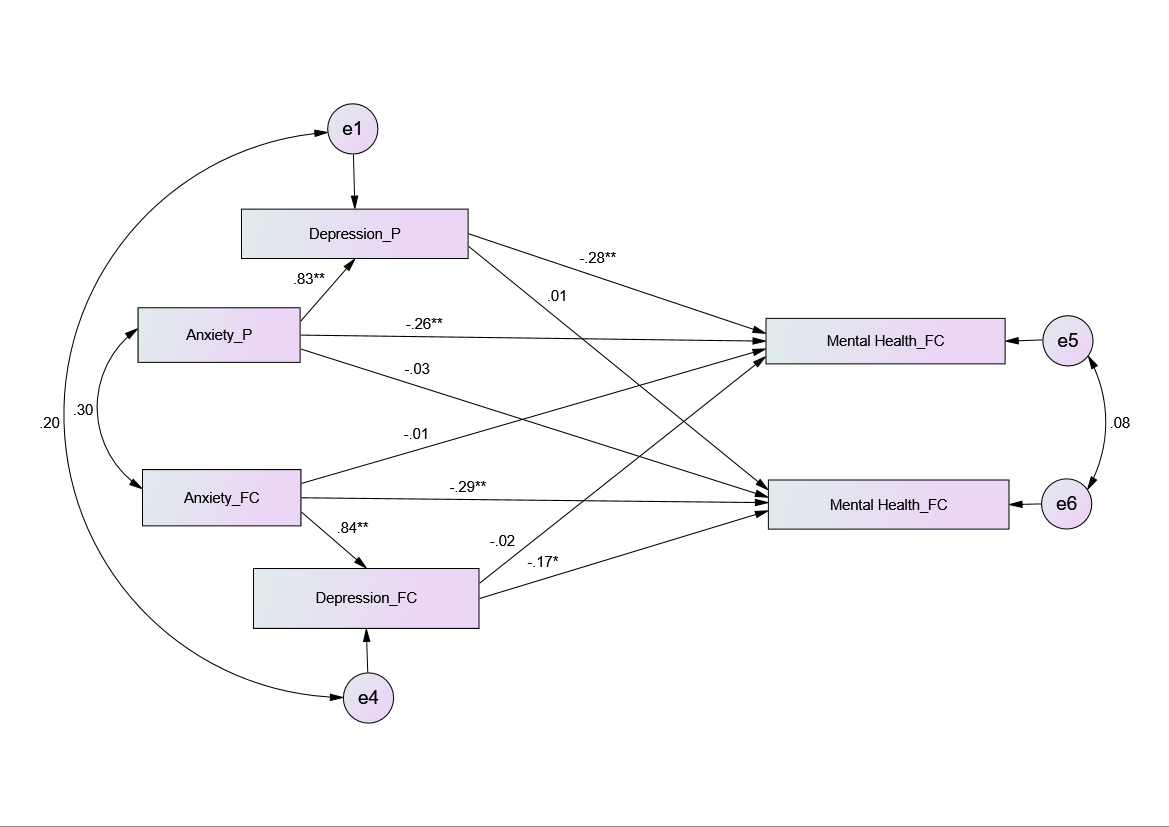


**P<0.05; **P<0.01*

*Sub-Model 10 Mental Health*
